# Cold Treatment Induces Transient Mitochondrial Fragmentation in *Arabidopsis* *thaliana* in a Way that Requires DRP3A but not ELM1 or an ELM1-Like Homologue, ELM2

**DOI:** 10.3390/ijms18102161

**Published:** 2017-10-17

**Authors:** Shin-ichi Arimura, Rina Kurisu, Hajime Sugaya, Naoki Kadoya, Nobuhiro Tsutsumi

**Affiliations:** 1 Graduate School of Agricultural and Life Sciences, The University of Tokyo, 1-1-1 Yayoi, Bunkyo-ku, Tokyo 113-8657, Japan; dandelion3409@yahoo.co.jp (R.K.); hjsugaya64@gmail.com (H.S.); kdynaoki@gmail.com (N.K.); atsutsu@mail.ecc.u-tokyo.ac.jp (N.T.); 2 Precursory Research for Embryonic Science and Technology (PRESTO), Japan Science and Technology Agency, 4-1-8, Honcho, Kawaguchi, Saitama 332-0012, Japan

**Keywords:** mitochondrial fission, dynamin, plant mitochondria, mitochondrial division

## Abstract

The number, size and shape of polymorphic plant mitochondria are determined at least partially by mitochondrial fission. Arabidopsis mitochondria divide through the actions of a dynamin-related protein, DRP3A. Another plant-specific factor, ELM1, was previously shown to localize DRP3A to mitochondrial fission sites. Here, we report that mitochondrial fission is not completely blocked in the Arabidopsis *elm1* mutant and that it is strongly manifested in response to cold treatment. Arabidopsis has an *ELM1* paralogue (*ELM2*) that seems to have only a limited role in mitochondrial fission in the *elm1* mutant. Interestingly, cold-induced mitochondrial fragmentation was also observed in the wild-type, but not in a *drp3a* mutant, suggesting that cold-induced transient mitochondrial fragmentation requires DRP3A but not ELM1 or ELM2. DRP3A: GFP localized from the cytosol to mitochondrial fission sites without ELM1 after cold treatment. Together, these results suggest that Arabidopsis has a novel, cold-induced type of mitochondrial fission in which DRP3A localizes to mitochondrial fission sites without the involvement of ELM1 or ELM2.

## 1. Introduction

Mitochondria are not made *de novo* but are created by fission of existing mitochondria [1]. The shape and number of higher plant mitochondria change drastically in response to changing environmental stimuli and changing developmental stages [2,3,4]. The shape and number of mitochondria are determined at least partially by the balance between mitochondrial fission and mitochondrial fusion. Frequent fission and fusion make it possible to share mitochondrial internal proteins and small molecules in each cell [5].

Mitochondrial fission is mediated by a type of GTPase called dynamin-related proteins (DRPs), which are well conserved in eukaryotes [6,7,8,9]. DRPs polymerize into a ring-like spiral structure surrounding mitochondrial fission sites from the outer surface of mitochondria, and then constrict to cleave the mitochondria by their GTPase activity [10,11,12,13]. Arabidopsis has 16 *DRP* genes. Two of them, *DRP3A* and *DRP3B* (formerly known as *ADL2a* and *ADL2b*), are most similar to mitochondrial fission-related DRPs in other eukaryotes [14,15]. DRP3A and DRP3B function redundantly and cooperatively in mitochondrial fission [15,16,17,18,19,20]. DRP3A seems to have a bigger role in mitochondrial fission than DRP3B. In T-DNA insertion mutants of *DRP3A* and *DRP3B* (*drp3a* and *drp3b*), mitochondria are longer and fewer in number than those in the wild type. Moreover, in *drp3a drp3b* double mutants, mitochondria are far more elongated, forming an interconnected network in each cell, because of the severe disruption of mitochondrial fission [19].

Arabidopsis has a plant-specific factor (ELM1) that localizes to the outer surface of mitochondria, where it interacts with DRP3A (and probably DRP3B) to localize them to mitochondrial fission sites [21]. In ethyl methanesulfonate (EMS)-induced and T-DNA insertion-induced *elm1* mutants, the mitochondria are elongated and fewer in number, suggesting that ELM1 is involved in mitochondrial fission [21].

Because the mitochondrial phenotype of *elm1* mutants is not as strong as that of *drp3a drp3b* double mutants, we hypothesized that residual mitochondrial fission occurs in the absence of ELM1. Arabidopsis has an ELM1 homologue of unknown function, ELM2, that is 54% identical (70% similar) to ELM1 at the amino acid sequence level. Here, we tested whether ELM2 is responsible for the residual mitochondrial fission in the absence of ELM1. During the course of this study, we also noticed that mitochondrial fission without ELM1 was transiently manifested by cold treatment. Therefore, we also examined whether transient cold-induced mitochondrial fragmentation needs ELM2 and DRP3A.

## 2. Results

### 2.1. Residual Mitochondrial Fission in the Mitochondrial Fission Mutant Elm1

**Figure 1:**
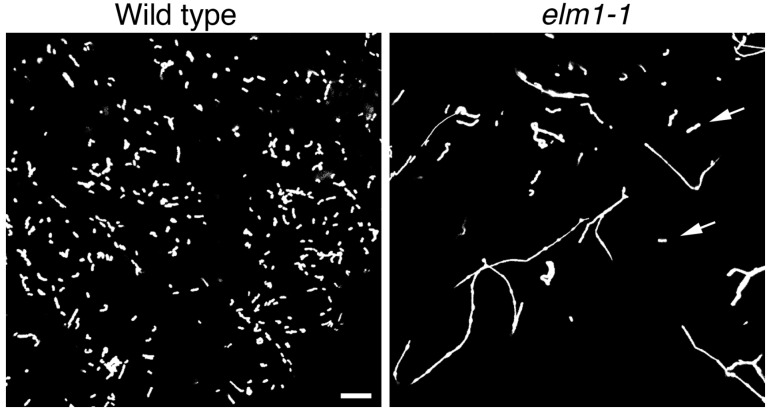
Mitochondrial morphologies in wild-type Arabidopsis and the *elm1-1* mutant. The images show GFP-labeled mitochondria in leaf epidermal cells. Mitochondria in the *elm1-1* mutant are longer and fewer than those in the wild type, because of the disturbance of mitochondrial fission in the mutant. However, *elm1-1* cells still have many short mitochondria (arrows), suggesting that mitochondrial fission can occur without ELM1. Scale bar, 10 μm, is applicable to the both figures.

The *drp3a drp3b* double mutant has a single interconnected mitochondrion in each cell because of the malfunction of mitochondrial fission without interruption of mitochondrial fusion [19]. Figure 1 shows representative micrographs of mitochondria in the wild type and *elm1-1* mutant. The latter has a point mutation that puts a termination codon in the middle of the ORF (open reading frame) [21]. Mitochondria in the *elm1-1* and other *elm1* allele mutants are longer and fewer in number than those in the wild type. Even in the mutants with the strongest phenotypes (*elm1-1* and *elm1-6*), each cell still has more than one mitochondrion and some of the mitochondria have particle shapes like those of the wild type (mitochondria indicated by arrows in Figure 1). These results suggest that mitochondrial fission is not completely blocked in the *elm1* mutants.

### 2.2. Is Mitochondrial Fission without ELM1 due to ELM2?

**Figure 2:**
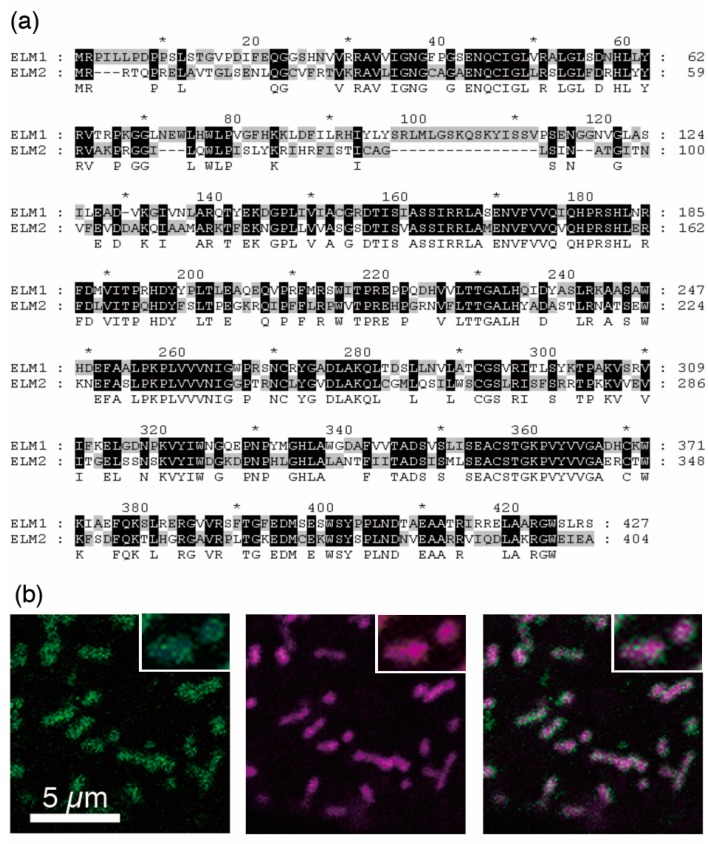
*ELM2* encodes an ELM1-like protein and GFP-tagged ELM2, like ELM1, localizes to the mitochondrial surface. (**a**) Clustal W alignment of ELM1 and ELM2 amino acids sequences. * depicts the positions of numbers in every ten amino acids. (**b**) Localization of GFP-ELM2 surrounding mitochondria. Arabidopsis cultured cells transiently expressing GFP-ELM2 with a mitochondrial marker MitoTracker were examined by confocal laser scanning microscopy (CLSM). A part of a single cell is shown. *Left* and *Center* are separate images obtained with the GFP and MitoTracker, respectively. *Right* is the merged image. Scale bar, 5 μm, is applicable to the other two figures. Upper right insets are X2 enlarged images.

The Arabidopsis genome has a single paralogue of *ELM1*, called *ELM2* (At5g06180). Its amino acid sequence is 54.0% identical (70% similarity, e-value 4.7 × 10^−117^) to that of *ELM1* (Figure 2a). The Arabidopsis genome had no other matches to *ELM1* (the next closest match had an e-value >0.1). When GFP: ELM2 was expressed under the CaMV35S promoter, the green signals seemed to surround the mitochondria (Figure 2b), as was the case with ELM1:GFP in our previous report [21]. This suggests that ELM2, like ELM1, localizes on the outer surface of the outer membrane of mitochondria.

**Figure 3:**
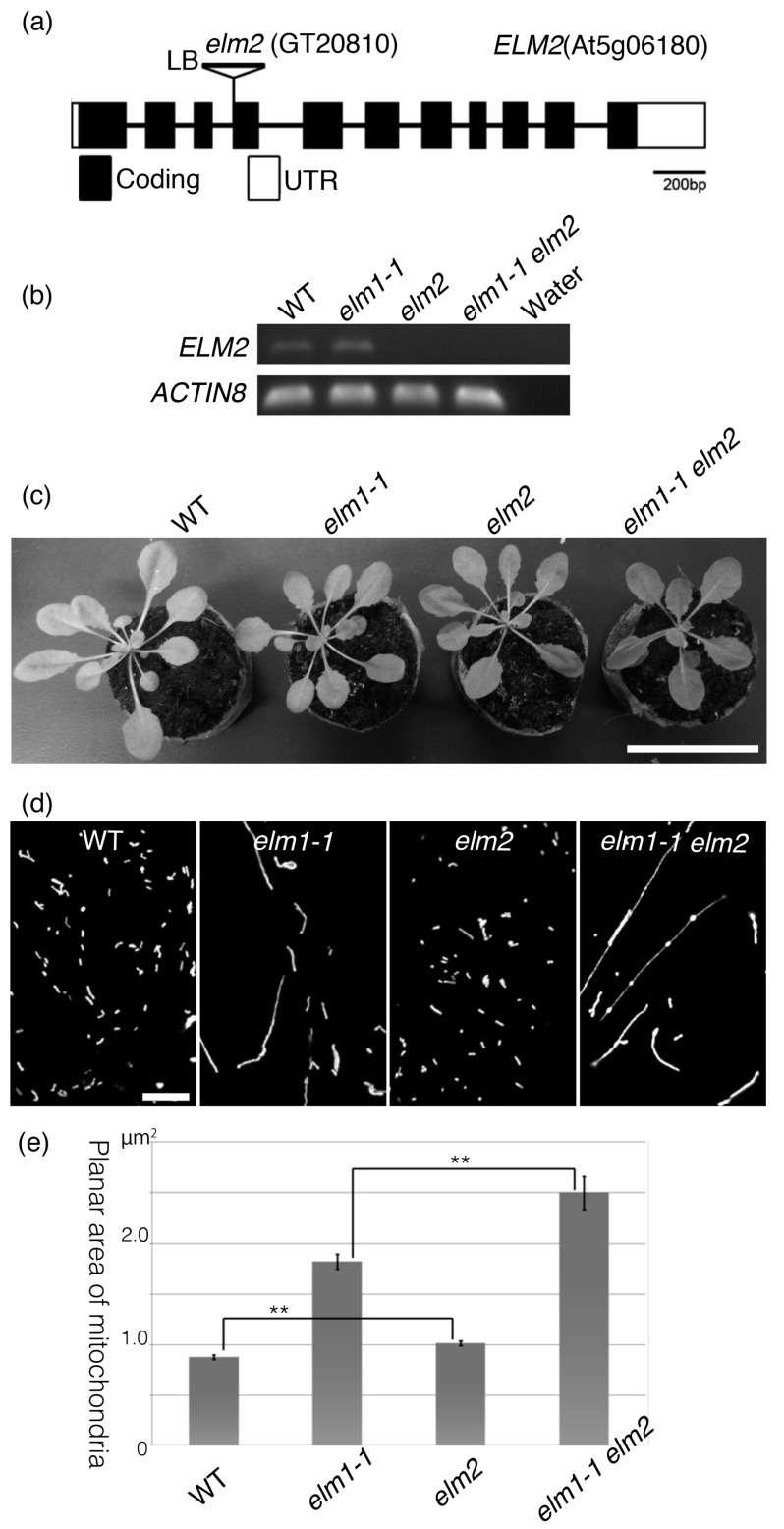
Disruption of *ELM2* does not appear to affect mitochondrial morphology much. (**a**) A T-DNA insertion in the end of the 3rd intron in the *elm2* mutant. (**b**) RT-PCR of full length of *ELM2* ORF (open reading frame) in the wild-type, *elm1-1*, *elm2* and *elm1-1 elm2* double mutants. (**c**) Comparison of growing phenotypes of wild-type, *elm1-1*, *elm2* and *elm1-1 elm2* double mutants. 30-day-old plants. Scale bar, 5 cm. (**d**) Mitochondrial morphologies in the wild-type, *elm1-1*, *elm2* and *elm1-1 elm2* double mutants. Leaf epidermal cells in 14-day-old plants were observed by confocal laser scanning microscopy. Scale bar, 10 μm, is applicable to the four images. (**e**) Average planar areas of mitochondria of wild type and mutants. (*n* > 218 in each of three replications) in each mutant. Error bars show S.E. ** indicates statistical significance at *p* < 0.01.

**Figure 4:**
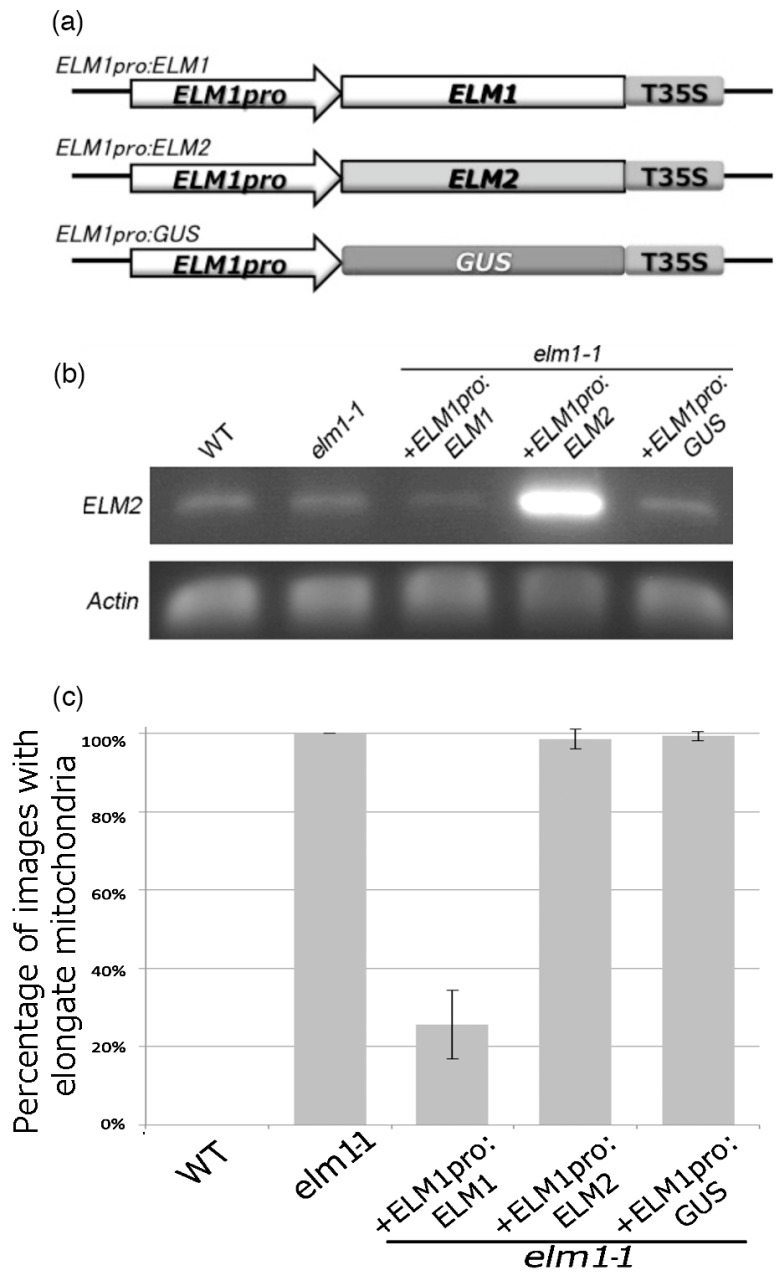
Heterologous complementation test of mitochondrial morphology in the *elm1* mutant by expression of *ELM2*. (**a**) Schematic drawing of DNA constructs used in this study. *ELM1*, *ELM2* and *GUS* coding sequences are attached between the probable promoter, the 950bp upstream region of *ELM1* and the sequence of CaMV35S terminator. (**b**) RT-PCR of the full length of the *ELM2* ORF in the wild-type, *elm1-1*, and three *elm1-1* mutants transformed with *ELM1pro:ELM1*, *ELM1pro:ELM2* and *ELM1pro: GUS* respectively. (**c**) Occurrence of elongate mitochondria in leaf epidermal cells in 14-day-old cotyledons from five Arabidopsis lines (wild-type, *elm1-1*, and three *elm1-1* mutants transformed with *ELM1pro:ELM1*, *ELM1pro:ELM2* and *ELM1pro: GUS*). Occurrence is expressed as the percentage of 40 confocal laser scanning microscopic images obtained from 8 leaves from each line that were judged to have elongated mitochondria (as in the *elm1-1* image in Figure 1). The experiments were repeated three times independently and the results were averaged. Error bars show S.E.

To test the possibility that the ELM2 functions in mitochondrial fission in the same manner as ELM1, the homozygous T-DNA insertion mutant *elm2* (Figure 3a) and the *elm1-1 elm2* double mutant were analyzed. An RT-PCR analysis (Figure 3b) shows that the *elm2* mutants did not accumulate full-length *ELM2* transcripts. The *elm1-1* mutants grew slightly more slowly than the wild type, as reported previously [21], but *elm2* grew almost as well as the wild type and the *elm1-1 elm2* double mutant grew almost as well as the *elm1-1* mutant. Similarly, the mitochondria in *elm2* were as small and numerous as those in the wild type, and the mitochondria in *elm1-1 elm2* double mutant were as long as those in the *elm1-1* mutant (Figure 3d). However, the average planar areas of mitochondria in the *elm2* and *elm1-1 elm2* double mutants were slightly but significantly larger than those in the wild type and *elm1-1* mutants, respectively (Figure 3e). These results suggest that ELM2 has a small effect on mitochondrial fission in the wild type and a small effect in the absence of ELM1. To test whether ELM2 complements ELM1, a chimeric sequence consisting of the *ELM2* ORF with the *ELM1* promoter (Figure 4a) was introduced into the *elm1-1* mutant. The *ELM1* promoter dramatically increased the expression of *ELM2* transcripts (Figure 4b) but did not rescue the mitochondrial fission defect in the *elm1-1* mutant (Figure 4c). This suggests that expression activity of the *ELM2* promoter is much weaker than that of the *ELM1* promoter and that *ELM2*, although a paralogue of *ELM1*, has much weaker activity than ELM1. Furthermore, cells of the *elm1-1 elm2* double mutant still had more than one mitochondrion and some of the mitochondria had particulate shapes (Figure 3d), suggesting that the mitochondria could divide without the involvement of either ELM1 or ELM2.

### 2.3. Transient Mitochondrial Fragmentation by Cold Treatment

**Figure 5:**
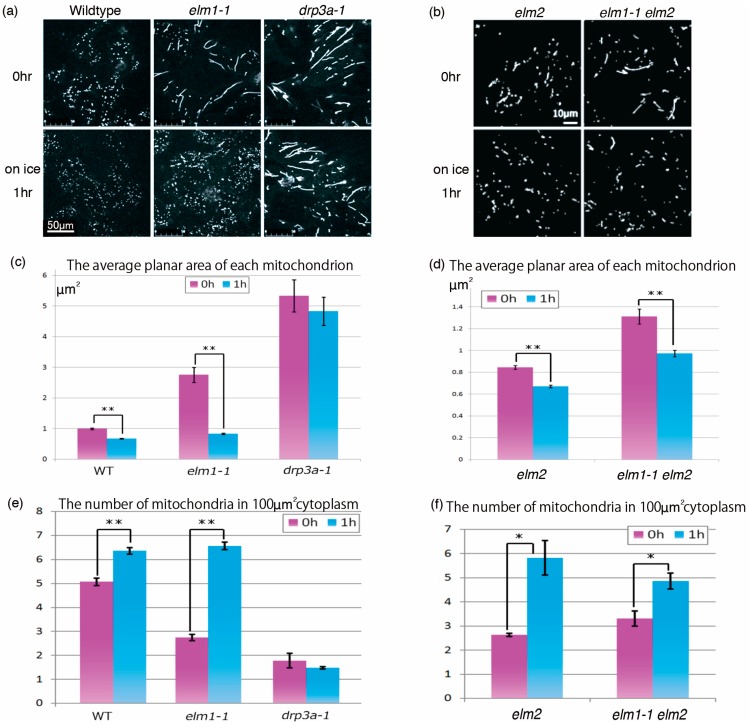
Mitochondrial fragmentation was induced by cold treatment in the wild-type and *elm* mutants but not in the *drp3a-1* mutant. (**a**,**b**), Representative mitochondrial morphologies in the wild type and mutants at room temperature before and 1 h after 4 °C treatment. Each scale bar is applicable to the all images in (**a**,**b**), respectively. (**c**,**d**) Average planar areas of mitochondria in epidermal cells of wild type and mutants before (red bars) and 1 h after (blue bars) cold-temperature treatment (*n* > 218 in each of three replications). (**e**,**f**) Average number of mitochondria per 100 μm^2^ in leaf epidermal cells of wild-type and mutants before (red bars) and 1 h after (blue bars) cold treatment. *n* = 3 Error bars show S.E. ** indicates statistical significance at *p* < 0.01 and * at *p* < 0.05. Because data sets (**a**,**c**,**e**) and (**b**,**d**,**f**) were collected independently in different conditions (e.g., laser strength, detector gain, etc.), they could not be compared with each other directly.

During the course of our observations, we noticed that cold treatment induced mitochondrial fragmentation in *elm1*. One hour at 4 °C increased the number and reduced the size of mitochondria, so that they became more like those of the wild type (Figure 5a). Such mitochondrial fragmentation was observed not only in the epidermal cells of leaves, but also in the epidermal cells of stems and roots (Figure S1). The light conditions in the cold treatment did not affect the mitochondrial fragmentation (data not shown). A similar morphological change was observed in *elm1-6*, a T-DNA insertion mutant (data not shown). Cold treatment decreased the area (Figure 5c) and increased the number (Figure 5e) of mitochondria, indicating that mitochondrial fission without ELM1 is manifested by cold in the *elm1-1* mutant. Because mitochondrial morphology is determined by the balance between fission and fusion, cold-induced mitochondrial fragmentation might be increased by down-regulation of mitochondrial fusion. However, whether or not fusion activity changes, mitochondrial fragmentation of the longer mitochondria requires mitochondrial fission. Interestingly, cold treatment also induced mitochondrial fragmentation in the wild-type but not in the *drp3a-1* mutant (Figure 5c,e). Cold also induced mitochondrial fragmentation in the *elm2* and *elm1-1 elm2* double mutants (Figure 5b,d,f). Together, these results suggest that cold-induced mitochondrial fragmentation in the wild type depends on DRP3A but not on ELM1 or ELM2.

**Figure 6:**
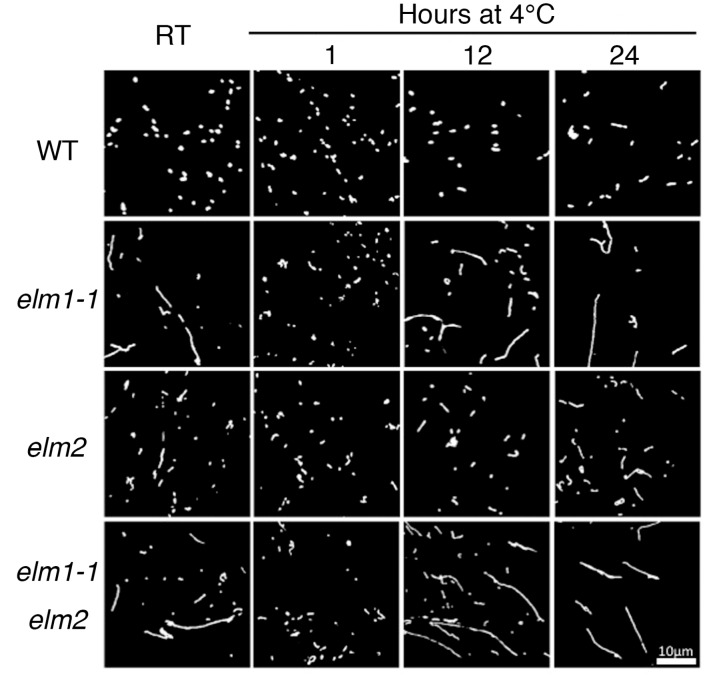
Mitochondrial morphology in the wild type and *elm* mutants after different durations of cold treatment. Mitochondria were observed in leaf epidermal cells of 28-day-old plants grown at 22 °C before and after different durations of 4 °C treatment. Scale bar, 10 μm, is applicable to the all images.

When the cold treatment was extended to 24 h, the number and shape of the mitochondria in the mutants reverted to their room temperature (22 °C) states (Figure 6), indicating that the cold-induced mitochondrial fragmentation is a transient phenomenon.

### 2.4. DRP3A Could Localize to Mitochondria without ELM1 at the Cold Treatment

**Figure 7:**
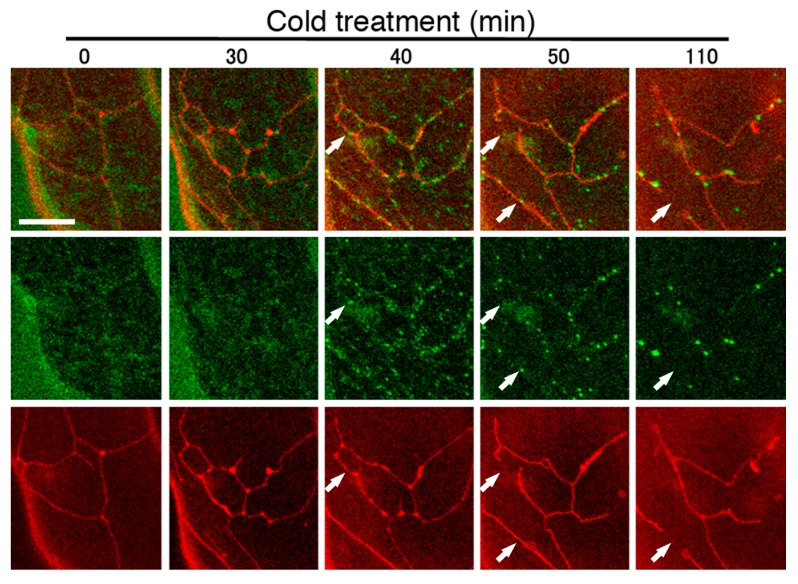
Time course observations of mitochondria and DRP3A in the *elm1* mutant. Images show a double-stained leaf epidermal cell of a 30-day-old *elm1-6* Arabidopsis plant transformed with DRP3Apro:DRP3A: GFP at different times after cold treatment. Bottom panels, mitochondrial network stained with MitoTracker; middle panels, DRP3AGFP; top panels, merged MitoTracker and GFP images. Cytosolic DRP3A: GFP first appeared as a hazy signal (0 and 30 min) and gradually localized and concentrated on mitochondria (40, 50 and 110 min). Arrows indicate sites of mitochondrial fission. Scale bar, 10 μm, is applicable to the all images.

We previously reported that the localization of cytosolic DRP3A to the mitochondrial fission sites required a functional ELM1 [21]. To confirm the present finding that ELM1 was not required for mitochondrial fission following cold treatment, we examined the behavior of DRP3A following cold treatment of *elm1-6* transformed with *DRP3A: GFP* driven by the DRP3A promoter*.* Before treatment (0 min in Figure 7), the mitochondria had an elongated network shape and the DRP3A: GFP signal was distributed in the cytosol, in agreement with our previous study [21]. DRP3A gradually appeared as small green particles in the cytosol and some of them localized on mitochondria at about 40 min after treatment (Figure 7). Subsequently, the intensity of the green dots increased on the mitochondria, and the intensity of cytosolic green signals decreased. At some locations (an example is shown by the arrows in Figure 7 at 40 and 50 min), the mitochondrial network divided at the green dots, suggesting that DRP3A served to divide the mitochondria at these sites. This result clearly shows that in response to cold treatment, DRP3A localizes from the cytosol to mitochondrial fission sites without ELM1.

## 3. Discussion

Mitochondrial fission occurs frequently to counterbalance the opposite event, mitochondrial fusion. In addition, mitochondria divide in accordance with cell division so that they are maintained in each of the daughter cells. In this study, we found that mitochondrial fragmentation can also be induced by cold treatment. However, we cannot rule out the possibility that downregulation of fusion was a contributing factor. Further studies are needed to test this possibility.

**Figure 8:**
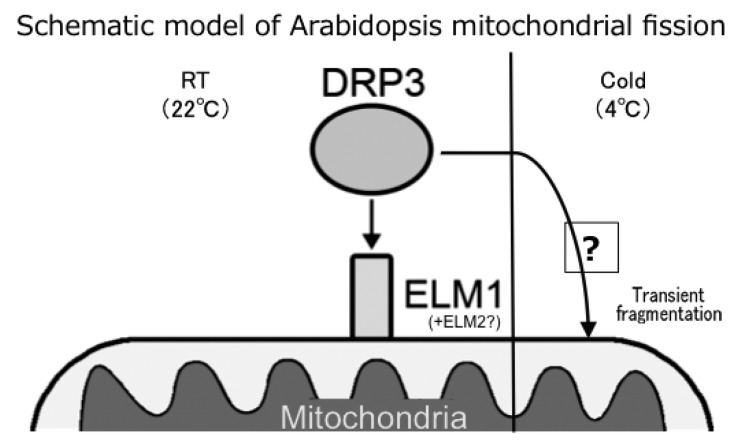
Schematic model of Arabidopsis mitochondrial fission. Two types of mitochondrial fission are drawn. In the normal condition (left, shown as RT (room temperature) 22 °C), the division executor, DRP3 localizes to mitochondria via interaction with ELM1. In the case of mitochondrial fission transiently induced by cold treatment, DRP3 could localize to mitochondria by skipping the help of ELM1 (and ELM2) and underwent fission.

Because the mitochondria in *elm1* mutants at room temperature (~22 °C) are usually very elongated (as in Figure 1), ELM1 is apparently important for mitochondrial fission in the wild type at room temperature, in which it localizes DRP3A to mitochondrial fission sites [21]. However, cold-induced fission does not involve either ELM1 or ELM2. ELM2 has only a limited role in mitochondrial fission (Figure 3 and Figure 5). This is further illustrated in the model shown in Figure 8. The finding that the *drp3a* mutant has similar elongated mitochondria at room temperature and 4 °C indicates that DRP3A is required for both types of mitochondrial fission. Mitochondrial fragmentation could be achieved by increasing fission or reducing fusion (or by both). Although we did not examine the effect of cold treatment on mitochondrial fusion, the present results confirm that cold-induced mitochondrial fission required the localization of DRP3A to the mitochondria.

The mechanism by which cold-treatment induced mitochondrial fission is unclear. The simplest idea is that the affinity between DRP3A and the mitochondrial outer membrane is transiently increased by the cold treatment. Purified DRPs in yeast and mammals bind to liposomes without any other proteins [10,13,22], although in vivo DRPs need other proteins to localize to mitochondrial fission sites from the cytosol. If cold treatment increases the affinity between DRP3A and the mitochondrial outer membrane, it would suggest that ELM1 is needed to support the binding of DRP3A to the outer membrane at room temperature but not at cold temperature due to the increased affinity of DRP3A at cold temperature. Further studies are needed to examine the effect of temperature on the affinity between purified DRP3A and the mitochondrial outer membrane.

Another possibility is that cold-induced mitochondrial fission involves other proteins. Tail-anchored proteins FIS1a (BIGYIN), FIS1b, PMD1 and PMD2 were also reported to be involved in mitochondrial division in *A. thaliana* [23,24,25]. However, none of them have been directly shown to have roles in the localization of DRP3A or DRP3B to the fission sites. PMD1 and PMD2 were shown to contribute to mitochondrial fission independent of DRP3/FIS1 [25]. In addition to protein components, a mitochondrial phospholipid (cardiolipin) was recently shown to stabilize the DRP3 complex on mitochondria [26]. These and unknown other proteins and lipid factors might contribute individually or together to the DRP3A localization and function in cold treatment or other types of mitochondrial fission. Furthermore, in *A. thaliana*, mitochondrial fission is reported to involve dynamin-related proteins other than DRP3A. These include DRP3B as well as the more distantly related DRP5B [20,27]. The relationships between the factors involved in mitochondrial fission, the different types of mitochondrial fission and how they are regulated appear to be more complicated than previously thought.

DRP3A was found to be phosphorylated and dephosphorylated at different stages of the cell cycle [28]. Proteomic analyses have predicted that DRP3A has multiple phosphorylation sites [29,30], but the effects of phosphorylation/dephosphorylation at these sites are unknown. Mammalian Drp1s, which are involved in mitochondrial fission, are also regulated by post-translational modifications other than phosphorylation, such as ubiquitination, SUMOylation and S-nitrosylation reviewed in [31]. Such modifications might also occur in plant DRPs. The overexpression of Arabidopsis UBP27, a mitochondrial outer membrane-bound ubiquitin protease, was recently reported to change mitochondrial morphology by inhibiting the binding of DRP3A and DRP3B to mitochondria, although it is unknown whether DRP3A and DRP3B are direct targets of UBP27 [32].

Plant mitochondria constantly undergo fission and fusion [5]. Such alterations appear to be involved in several activities that are crucial to the health of cells [33,34]. It is unclear what processes may be involved in cold-induced mitochondrial fragmentation in Arabidopsis, although because cold adaptation affects the expression of over 2000 genes in Arabidopsis [35], there are many candidates. Many of these genes are expressed days and weeks after cold treatment, whereas mitochondrial fragmentation occurs within an hour, indicating that it is one of the early responses to cold treatment. In mammalian brown adipose tissue, cold exposure induces thermogenesis, which has been linked to mitochondrial fragmentation through activation of a DRP3A homologue [36]. However, cold stress does not seem to induce mitochondrial thermogenesis through uncoupling proteins in Arabidopsis [37,38]. Further studies are needed to see which of the many metabolic changes in cold stress are responsible for mitochondrial fragmentation in Arabidopsis.

The balance between mitochondrial fission and fusion appears to vary in different tissues and in different environmental conditions in order to change mitochondrial morphology to meet the cells’ physiological needs. The shape, distribution and number of mitochondria change in accordance with organ development [2,4,39] and in response to environmental stimuli [3,40]. Changes of mitochondrial morphology, numbers and distribution would affect the three-dimensional distances and attachments between mitochondria and other organelles metabolically related to mitochondria, causing indirect effects on cell metabolisms and physiological states [41]. Thus, a better understanding of the mechanisms underlying the changes in mitochondrial morphology should help to clarify a number of cellular processes in plants.

## 4. Materials and Methods

### 4.1. Plant Materials and Growth Conditions

*Arabidopsis thaliana* ecotype Columbia (Col-0) and its transformant with mitochondrial-targeted GFP [42] were used as wild-type plants in this paper. The EMS mutants *elm1-1* and *drp3a-1* were described previously [21]. All *Arabidopsis* plants were grown in growth chamber at 22 °C under a 14 h photoperiod at 50~100 μmol/m^2^s. The T-DNA insertion line GT20810 was provided by the Cold Spring Harbor Laboratory (http://www.cshl.edu/). GT20810 was consecutively crossed with Col-0 5 times to obtain a background similar to that of Col-0. The homo-T-DNA insertion line of the BC5F2 was used as *elm2*. The T-DNA insertion was checked by PCR with primers 1 and 2 to detect WT DNA and primers 1 and 3 to detect the T-DNA insertion. The homozygous and heterozygous *elm1-1* point mutations were checked by sequencing and PCR with primers 4 and 6 to detect the mutated DNA and primers 5 and 6 to detect the wild-type DNA. The primers are shown in Table S1.

### 4.2. Construction of Plasmids

*ELM2* ORF was obtained by RT-PCR from *A. thaliana* col-0 RNA with primers 3 and 15 and cloned into pENTR^TM^/d-TOPO entry vector (Invitrogen). The Ti plasmid expressing GFP: ELM2 fusion protein was constructed by LR reaction of Gateway cloning technology (Invitrogen) with pH7WGF2 destination vector, which was kindly provided from VIB [43]. An In-Fusion HD cloning kit (TaKaRa) was used to make Ti plasmids for expressing *ELM1*, *ELM2* and *GUS* under the *ELM1* promoter. The promoters consisted of 950 bp of the region upstream of the ATG initiation codon of *ELM1*. The promoter was amplified from genomic DNA and the ORFs were amplified by RT-PCR. The basal Ti-plasmid was pBGWFS7 [43]. Oligonucleotide primers are presented in Table S1 and the combinations of primers to make constructs are presented in Table S2. All PCR for DNA construction was carried out with high-fidelity DNA polymerases. All constructs made in this study were confirmed by sequencing. The T-DNA insertion line *elm1-6* transformed with *DRP3Apro:DRP3A: GFP* used in Figure 7 was previously described [21].

### 4.3. Agrobacterium Mediated Transformation of Arabidopsis Plants and Cultured Cells

The Ti plasmids described above were transformed into *Agrobacterium tumefaciens* strain C58C1. The Arabidopsis plants in Figure 4 were transformed with *A. tumefaciens* via floral dipping [44]. Transgenic T1 plants were selected on the MS-Agar medium containing 35 mgL^−1^ glufosinate-ammonium (Sigma-Aldrich). The Arabidopsis transgenic cultured cells used in Figure 2b were made as follows. The transformed *Agrobacterium* was cultured in LB medium containing 50 mg L^−1^ hygromycin and 100 mg L^−1^ spectinomycin (O.D. = 0.5 at 600 nm), pelleted and re-suspended in modified MS medium and used to inoculate 10 ml culture of 2-day-old Arabidopsis Col-0 suspension-cultured cells, called Alex. To remove *Agrobacterium*, 30 μL of 250 mg mL^−1^ claforan was added to the culture medium at 1 day after inoculation. The Arabidopsis cells were transferred to fresh media 5 days after the inoculation, and they were observed by microscopes 8 days after the *Agrobacterium* inoculation.

### 4.4. MitoTracker Orange Staining

The suspension-cultured transformed cells in Figure 2 were stained with 50 μM MitoTracker Orange (Molecular Probes) for 30 min and washed with medium three times. In the experiment in Figure 7, small sections (10~50 mm^2^) were cut out from the Arabidopsis leaves with new razor blades and stained with 50 μM MitoTracker Orange (Molecular Probes) for about 60 min.

### 4.5. Microscopic Observations and Image Analysis

A confocal laser scanning microscope (CLSM) (Nikon TE2000-U and C1Si) was used for all microscopic observations of Arabidopsis leaves and cultured cells with fluorescent fusion proteins or stained with fluorescent dyes. Fluorophores of GFP and MitoTracker Orange were excited by A 488 nm and A 561 nm laser, respectively. Emission signals were detected through a 515/30 nm filter for GFP and a 590/70 nm filter for MitoTracker Orange. All CLSM images were acquired in single focal planes. The acquired images were prepared with Photoshop CS5 (Adobe Systems) and analyzed with Image pro plus 4.0 (Media Cybernetics). The averaged mitochondrial number in every 100 μm^2^ microscopic observation area and the averaged area of each mitochondrion were measured and calculated with Image-Pro Plus ver.6.2J (Media Cybernetics) from the CLSM images before and after the cold treatment (Figure 5c–f).

### 4.6. RT-PCR Analysis

Total RNA for RT-PCR analysis was extracted from about one-month-old Arabidopsis leaves by using an RNeasy plant mini kit (Qiagen) according to the manufacturer's instructions; 400 ng of total RNA were used for RT-PCR analysis. Reverse transcription was carried out with Oligo-dT primer and the Super Script III reverse transcriptase (Invitrogen), and amplified with KOD FX Neo polymerase (TOYOBO). PCR was done with the specific primers 3 and 15 for *ELM2* and 16 and 17 for *ACTIN8* presented in Table S1.

### 4.7. Cold Treatment

The plantlets and samples on glass slide were incubated in 4 °C incubators. The plantlets were illuminated with a desk-top light with similar strength. To obtain the successive images of single cells under cold treatment in Figure 7, a small petri dish containing cold water and ice was placed on the slide glass on an inverted microscope (Nikon TE2000-U).

## Supplementary Materials

Supplementary materials can be found at www.mdpi.com/1422-0067/18/10/2161/s1.

## Abbreviations

DRP	Dynamin-related protein
ELM1	Elongate mitochondria
