# Supplementary material for: Cold Treatment Induces Transient Mitochondrial Fragmentation in Arabidopsis thaliana in a Way that Requires DRP3A but not ELM1 or an ELM1-Like Homologue, ELM2
Source: Int J Mol Sci. 2017 Oct 17;18(10):2161. doi: 10.3390/ijms18102161 (PMC5666842; doi:10.3390/ijms18102161)
Supplement: Supplementary file 1 [file ijms-18-02161-s001.pdf]

Supplementary Materials: Cold Treatment Induces Transient Mitochondrial Fragmentation in *Arabidopsis thaliana* in a Way that Requires DRP3A but not ELM1 or an ELM1-Like Homologue, ELM2

Shin-ichi Arimura, Rina Kurisu, Hajime Sugaya, Naoki Kadoya and Nobuhiro Tsutsumi

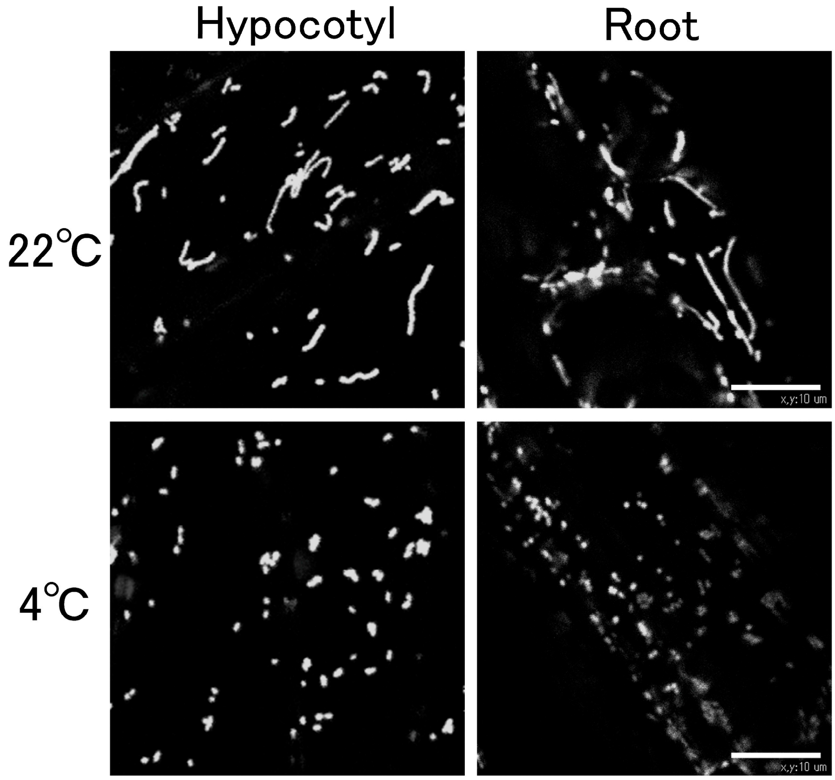

Figure S1. Mitochondrial fragmentation was induced by cold treatment in the epidermal cells of hypocotyl and root of the 70-day-old *elm* mutants. The images show GFP-labeled mitochondria. Scale bar, 10µm.

Table S1. Primer List（All directions are from 5′ to 3′)

Primer 1	ACGGTCGGGAAACTAGCTCTAC
Primer 2	GGATGTTCACGTGGAGTTAC
Primer 3	ATGCGACGAACACAGCCACG

Primer 4	CCCTAGATTTATGCGTAGTTTAA
Primer 5	GCCTCAGTGTTCAAAGAATATGG
Primer 6	CCCTAGATTTATGCGTAGTTTGA
Primer 7	CGGCCATGCTAGAGTCCGCA
Primer 8	CATATGGGAGAGCTTGCATG
Primer 9	AAGCTCTCCCATATGCACCTTAACGATTCTTGTCT
Primer 10	ACTCTAGCATGGCCGTCAAGACCGTAAACTCCATC
Primer 11	GACGGCCAGTTCCCGTGGCTGTGTTCGTCGCATGCCGGTTAGATTATCGA
Primer 12	ACTCTAGCATGGCCGTCATGCCTCGATTTCCCAGC
Primer 13	ATGTTACGTCCTGTAGAAAC
Primer 14	TTCTACAGGACGTAACATGCCGGTTAGATTATCGA
Primer 15	GCCGGTCAAAGGTCGAACAG
Primer 16	ACATCAAGGAGAAGCTTTCC
Primer 17	CCGTCATGGAAACGATGTCT

Table S2. Primer combinations for making each construct

Construct Names	PCR for vectors		Inserts	
	Forward	Reverse	Forward	Reverse
ELM1pro:ELM1	Primer 7	Primer 8	Primer 9	Primer 10
ELM1pro:ELM2	Primer 7	Primer 8	Primer 9	Primer 11
			Primer 3	Primer 12
ELM1pro:GUS	Primer 13	Primer 8	Primer 9	Primer 14
